# Automatic diagnosis of depression based on attention mechanism and feature pyramid model

**DOI:** 10.1371/journal.pone.0295051

**Published:** 2024-03-12

**Authors:** Ningya Xu, Hua Huo, Jiaxin Xu, Lan Ma, Jinxuan Wang

**Affiliations:** 1 Information Engineering College, Henan University of Science and Technology, Luoyang, Henan, China; 2 Engineering Technology Research Center of Big Data and Computational Intelligence, Henan University of Science and Technology, Luoyang, Henan, China; BMS Institute of Technology and Management, INDIA

## Abstract

Currently, most diagnoses of depression are evaluated by medical professionals, with the results of these evaluations influenced by the subjective judgment of physicians. Physiological studies have shown that depressed patients display facial movements, head posture, and gaze direction disorders. To accurately diagnose the degree of depression of patients, this paper proposes a comprehensive framework, Cross-Channel Attentional Depression Detection Network, which can automatically diagnose the degree of depression of patients by inputting information from the facial images of depressed patients. Specifically, the comprehensive framework is composed of three main modules: (1) Face key point detection and cropping for video images based on Multi-Task Convolutional Neural Network. (2) The improved Feature Pyramid Networks model can fuse shallow features and deep features in video images and reduce the loss of miniscule features. (3) A proposed Cross-Channel Attention Convolutional Neural Network can enhance the interaction between tensor channel layers. Compared to other methods for automatic depression identification, a superior method was obtained by conducting extensive experiments on the depression dataset AVEC 2014, where the Root Mean Square Error and the Mean Absolute Error were 8.65 and 6.66, respectively.

## Introduction

Major depressive disorder (MDD) is a mood dysfunction characterized by persistent spontaneous depressed mood, mainly caused by abnormalities in the genetic system of the patient or by drastic changes in the acquired environment. According to the World Health Organization (WHO), approximately 350 million people worldwide are predisposed to depression, of which 280 million have been diagnosed, including 5.0% of adults and 5.7% of people over 60 years of age worldwide [1]. The typical clinical manifestations of depression include low mood, slowed thinking, and irritability, while some people even experience insomnia, loss of appetite, dizziness, and fatigue. More severe patients may be suicidal, with more than 700000 depressed people heading for suicide each year, the second leading cause of death among people aged 15-29 [2]. Fortunately, depression can be alleviated with appropriate medication, psychological reassurance, and several other clinical treatments. Currently, most diagnoses of depression are evaluated by medical professionals, with the results of these evaluations influenced by the subjective judgment of physicians. In recent years, the early diagnosis and reassessment of follow-up treatment effects have been limited by the increase in depression patients. Therefore, to provide a more accurate diagnosis of depression levels, many depression detection methods based on machine learning have been developed with a wide scope of applications for objective and rapid diagnosis.

With the development of machine learning technology, machine learning has achieved remarkable achievements in the fields of computer vision, natural language processing, and speech recognition. Machine learning techniques have been applied to the early identification of depression and have attracted the attention of many research scholars. The automatic diagnosis of depression could be performed in four main ways: (1) Text-based semantic analysis method [3–5]. (2) Speech-based acoustic feature analysis method [6–8]. (3) Video-based facial expression analysis method [9–11]. (4) Electroencephalogram (EEG) signal-based data analysis method [12–14]. Dinkel et al. [3] proposed a text-based multi-task BGRU network with pre-trained word embeddings for the responses of simulating patients in clinical interviews, demonstrating that pre-trained words are effective for depression detection. Ma et al. [8] proposed a deep model termed DepAudioNet, which combines Convolutional Neural Network (CNN) and Long Short-Term Memory Neural Network (LSTM) to encode depression-related features in speech, achieving the purpose of detecting depression. Niu et al. [9] proposed a model for depressed patients, by processing facial changes through Graph Convolution Embedding (GCE) blocks and Multi-Scale Vectorization (MSV) blocks to predict depression levels. Jiang et al. [13] proposed a Task-related Common Spatial Pattern (TCSP) for detecting EEG in depressed patients using spatial information, and evaluated different classifiers finding that the use of TCSP improved the performance of the classifier and enhanced the spatial discrepancy before feature extraction.

Since depressed individuals exhibit disturbed facial movements, head posture, and gaze direction, facial expression features are essential for identifying depression and can convey evidence of depressed states [15]. The typical sad or neutral expression is observed in depressed patients, with frequent manifestations of fatigue, anxiety, and reduced socialization levels (e.g., reduced facial activity, avoidance of eye contact, decreased smiling, etc). The study by psychologist Mehrabian [16] demonstrated that 55% of depressive traits can be expressed from changes in facial expressions, 38% from the voice, and only 7% from the content of speech. Therefore, changes in facial expressions can be considered biomarkers of depression severity and can be estimated using the Beck Depression Inventory-II (BDI-II) score [17], which was shown in Table 1.

**Table 1:** BDI-II scores and corresponding depression severity.

BDI-⨿⋅Score	Depression⋅Severity
0-13	None
14-19	Mild
20-28	Moderate
29-63	Severe

Based on these, a comprehensive framework for automatic diagnosis of depression based on visual feature analysis of facial regions, the Cross-Channel Attentional Depression Detection Network (CCANet), was proposed in this study. In this experiment, the iconic depression features and final labels of depressed patients were extracted from the given videos as training inputs, and the given depression labels were used to predict the depression level of other patients, The effectiveness of the proposed method was confirmed by experimental results on the dataset AVEC 2014 [18]. In particular, there are three main contributions:

(1) A new framework for analyzing depression based on facial region features was proposed, which effectively utilizes facial features to automatically detect the degree of depression.
(2) The FPN model is introduced to fuse shallow and deep features of facial expression data in depression recognition.
(3) A cross-channel attentional convolutional neural network (CCA-CNN) is proposed, which was added to the FPN model to enhance the interaction between the tensor channels obtained from the convolutional layers.

## Related works

Generally, researchers believe that the manifest characteristics of depression can be identified by a large number of visual signals [19], such as involuntary changes in facial action units (AU) [20–22], eye gaze direction [23–25], pupil dilation response [9, 26, 27], facial expressions [28, 29] and head movement posture [30]. These biomarkers effectively capture mental disorders caused by depression and represent critical signals for the automatic detection of depression. Additionally, visual behavior is more complex and variable compared to textual and speech features, and thus, it is more challenging to capture depression-related cues through visual behavioral features. In this section, we briefly review previous research efforts in depression identification.

For visual cues, there are two main techniques for automatic depression recognition methods: hand-crafted descriptor-based approach and deep learning-based feature extraction approach, which has yielded positive results in the field of automatic depression recognition.

### Hand-crafted descriptor-based approach

Meng et al. [31] first used a Motion History Histogram (MHH) to extract dynamic features from corresponding video and audio to characterize subtle changes in the faces and voices of depressed patients. The Partial Least Squares (PLS) regression algorithm was performed and the relationship between dynamic features and depression scales was explored with training data to predict unknown depression scales. In addition, Cummins et al. [32] performed alignment of each video file to obtain face regions and extract Space-Time Interest Points (STIP) and Pyramid of Histogram of Gradients (PHOG) features. Subsequently, k-means clustering was performed on STIP and PHOG, and histograms were generated by computing Bag-of-Words (BoW). Finally, these visual features were trained and tested with Support Vector Regression (SVR) to determine the BDI scores of the subjects. The experimental results demonstrated that the PHOG feature showed superior test results than the STIP feature. Jan et al. [33] proposed a generation method based on the MHH idea of extracting dynamic features in the video feature space for extracting two-dimensional motion features in videos. Then, the relationship between dynamic features and depression scales is explained by PLS and linear regression methods. Finally, feature fusion is performed for predictions from video and audio patterns while validating the effectiveness of its method on the AVEC 2014 dataset. Kaya et al. [24] obtained regression results by processing the extracted histogram and Local Phase Quantization (LPQ) features of Local Gabor Binary Patterns from Three Orthogonal Planes (LGBP-TOP) using Canonical Correlation Analysis (CCA) and Moore-Penrose Generalized Inverse (MPGI) for the study of the facial regions corresponding to the eyes and mouth. Wen et al. [34] extracted the dynamic feature descriptor LPQ from the tri-orthogonal plane of the facial region subvolumes and then composed all LPQ vertices into a descriptor of nonverbal behavioral modality using both sparse matrix encoding and discriminative mapping and finally SVR was used to train and predict the features to further improve the accuracy of diagnosis. He et al. [35] combined the Median Robust Local Binary Pattern (MRLBP) with the TOP framework to obtain Median Robust Local Binary Patterns from Three Orthogonal Planes (MRLBP-TOP) features with strong robustness for capturing minor changes in facial expressions and time-domain information of macrostructures in video images. Niu et al. [36] proposed a new Local Second-Order Gradient Cross Pattern (LSOGCP) for extracting subtle facial texture features. The video features represented as LSOGCP-TOP are formed by generating LSOGCP histograms from tri-orthogonal planes.

### Deep learning-based feature extraction approach

As deep learning techniques continue to mature, Recurrent Neural Networks (RNNs), Convolutional Neural Networks (CNNs), and Long-Short Term Memory networks (LSTMs) have been demonstrated to be effective in detecting depression in the early diagnosis of depression. Zhu et al. [37] introduced Deep Convolutional Neural Networks (DCNNs) to the automatic depression recognition task and designed a dual-stream framework to capture the facial appearance and dynamic features with training analysis of deep convolutional neural networks to predict the BDI-II scores of depressed patients. Zhou et al. [38] proposed a deep convolutional network termed DepressionNet, which employs a Global Average Pooling layer (GAP) to process video frames and judge the depression severity according to the generated Depression Activation Map (DAM), and based on these, a multi-region depression network is proposed, which can jointly learn multiple regions of a face and fuse their responses to improve the overall recognition performance. Al Jazaery et al. [39] proposed a new framework for automatically learning spatiotemporal features of face regions at two different scales using Three-Dimensional Convolutional Neural Networks (3D-CNN), and then inputting sequences of spatiotemporal information sequences into RNNs to predict the degree of depression by processing closely aligned and loosely unaligned face features. Zhou et al. [40] adopted an attentional mechanism to learn deeply discriminative representations of depression, which introduced a pooling layer to vary facial image weights for different poses and imaging conditions by adaptive learning, however, the mechanism only considered the spatial dependence of the extracted features while ignoring the temporal information between video frames. de Melo et al. [41] integrated a 3D global averaging pool to improve the ability to extract spatiotemporal features, and then extracted spatiotemporally relevant features from the full face and localization of the test patient by fusing multiple 3D convolutional neural networks, however, no fully connected layer was applied to reduce the parameters of the model to prevent overfitting. Song et al. [25] proposed a multiscale video-level descriptor and introduced spectral heatmaps and spectral vectors to learn the representation of visual features and input the spectral representation to CNNs and Artificial Neural Networks (ANNs) for training and analysis. Uddin et al. [42] used the Inception-ResNet-v2 network to capture facial motion information, after which the obtained facial feature information was fed into the CNN to obtain more discriminative features, and the authors introduced the LSTM model to obtain temporal information by integrating the Temporal Median Pool (TMP) into the model. Hao et al. [43] introduced Bidirectional Long and Short-Term Memory Neural Networks (Bi-LSTMs) for extracting contextual temporal information of facial features and text features and attention mechanism for learning the relationship between text features and facial feature morphology in a deep learning algorithm to perform depression recognition by combining text features and facial features. He et al. [11] proposed a comprehensive framework for automatic depression diagnosis based on video face image sequences, which used multiple pre-trained models to represent low-level features and captured high-level feature information for depression analysis using a feature aggregation module and confirmed their findings on the AVEC 2013 and AVEC 2014 datasets.

## Automatic prediction of depression based on CCANet network

**Fig 1:**
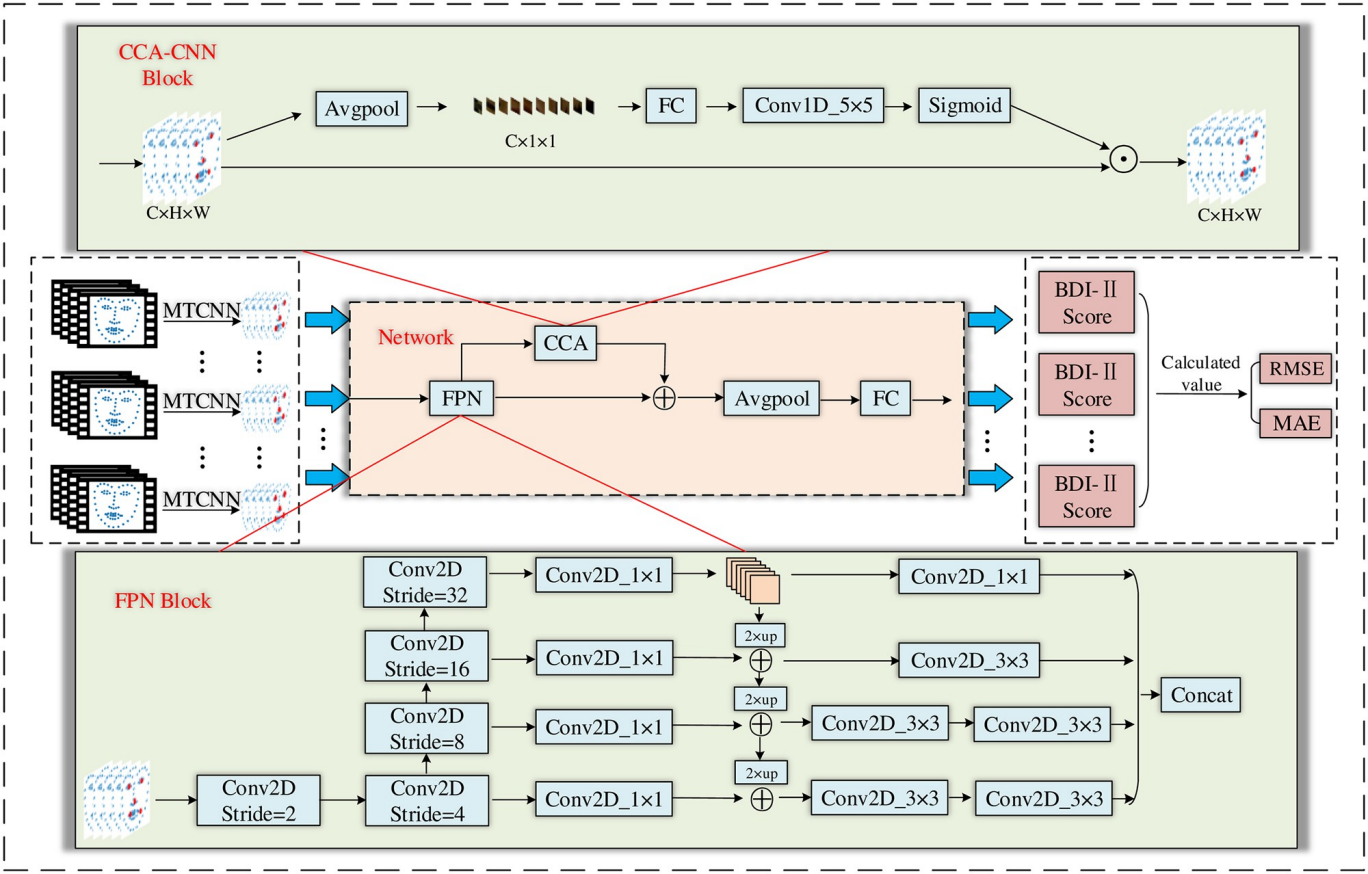
The CCANet framework flowchart based on attention mechanism and feature pyramid model. CCA-CNN module represents the cross-channel attention convolutional neural network. FPN module refers to the modified FPN model. The ‘⊕’ indicates that the features are fused by summation and the ‘⊙’ denotes the phase multiplication.

In this section, we initially describe our proposed 2D-CNN architecture based on CCANet, as is shown in Fig 1. To combine the feature information of different size layers in the image, we improve the FPN model by fusing the shallow features and deep features of the face image to be detected and propose the CCA module to enhance the interaction between the tensor channels obtained from the convolutional layers. Firstly, preprocessing is performed. The detected video is extracted to obtain video frames, followed by inputting the video frames into MTCNN [44] to recognize faces cropping the video frames to obtain face images, and then regressing the obtained face images and outputting the key point coordinates. Subsequently, feature extraction is performed. The face images with key point coordinates are input to the FPN [45] module for the extraction and fusion of face features, and then the extracted features are input to the CCA module for convolutional fusion to enhance the representation of image features. Finally, the obtained image features are processed through the average pooling layer and the fully connected layer, and then the RMSE and MAE are obtained by calculation. We conducted our experiments on the AVEC 2014 standard dataset and obtained RMSE = 8.65 and MAE = 6.66. The comparison revealed that our experiments demonstrated superior performance in the depression detection task.

### Multi-Task Convolutional Neural Network (MTCNN)

**Fig 2:**
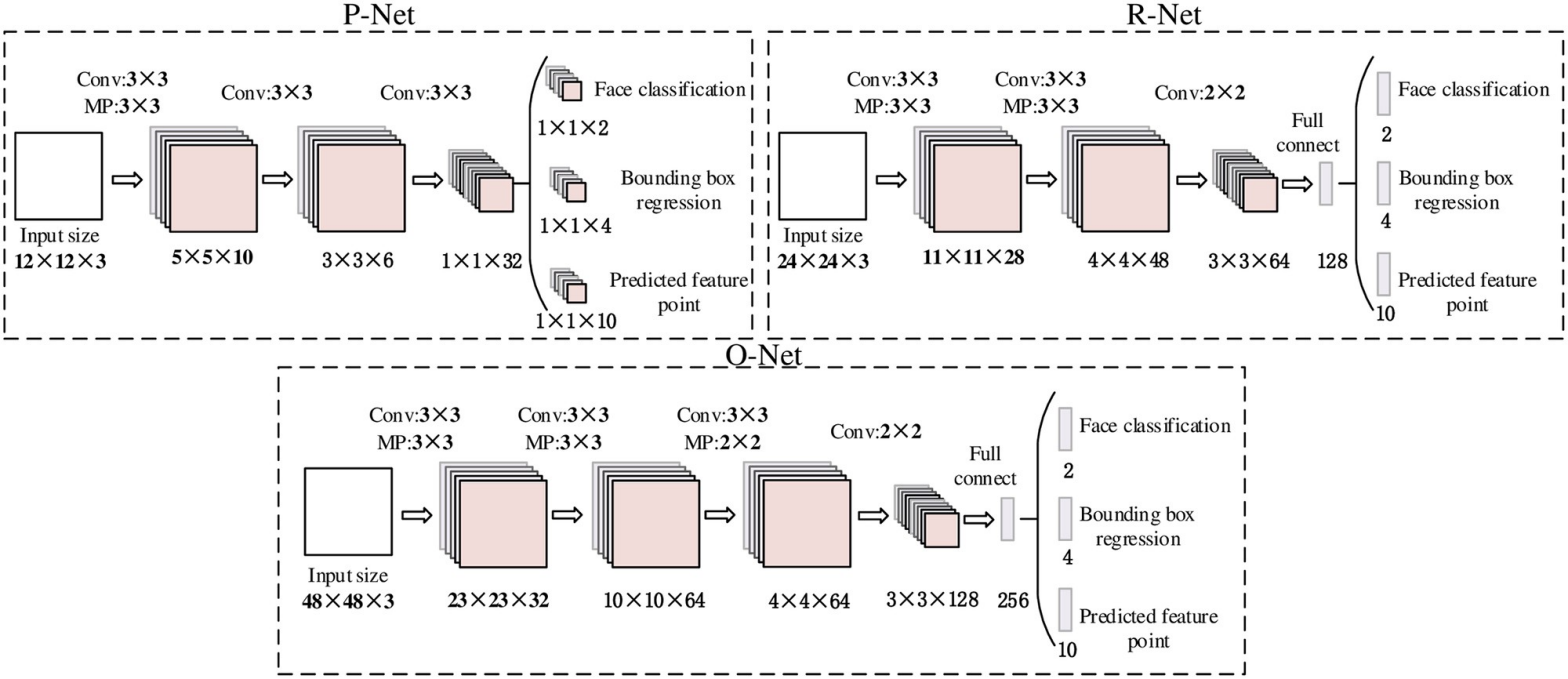
The architectures of P-Net, R-Net and O-Net. Where “MP” means max pooling and “Conv” means convolution. The step size in convolution and pooling is 1 and 2, respectively.

Eisenbarth, H. et al. [46] confirmed the relevance of eyes and mouth for emotion decoding by using an eye-tracking method to monitor scanning behavior in healthy subjects while observing different facial expressions and calculating the dominance ratios of eyes and mouth relative to other facial behaviors, finding that changes in the characteristics of the eyes and mouth were more noticed in sad and depressed facial expressions. Given that most previous face detection algorithms use a CNN model that separates face detection and alignment, the correlation between the two tasks is ignored. To exclude the effect of correlation between tasks and improve the accuracy of the model, this experiment uses the MTCNN approach to perform fast and accurate face detection based on the idea of adding classifiers to candidate frames. As shown in Fig 2, the MTCNN scales the image into different sizes based on different adjustment factors to form a feature pyramid of the image. Next, there are three stages consisting of three cascaded CNN network structures, that is, the Proposal Network (P-Net), the Refinement Network (R-Net), and the Output Network (O-Net).

The P-Net performs initial boundary calibration of the image pyramid using a Fully Convolutional Network (FCN) to generate a large number of candidate windows and boundary regression vectors and uses convolutional kernel sliding for multiple extractions of the original image, which can accurately calibrate the approximate position of the target subject (the face in the image). The candidate windows are border-calibrated according to the bounding box and overlapping windows are removed using Non-Maximum Suppression (NMS). After that, the candidate windows identified by P-Net are adjusted with high precision using R-Net, and the face in the image is targeted, the candidate windows are adjusted using boundary regression vectors, and then overlapping windows are removed by using NMS. The O-Net network generates a calibrated frame of the face region that meets the requirements, overlapping windows are removed using NMS, and then the output of five facial feature points is obtained after coordinate regression on the face region.

The MTCNN feature descriptor mainly consists of three parts, which are Face classification, Bounding box regression, and Predicted feature points. Face detection is a binary classification task and thus less number of filters are required, setting 3 × 3 filters in the MTCNN algorithm reduces the amount of data to be computed while increasing the depth for better performance.

The face classification is performed on the extracted video frames, with the cross-entropy loss function used for this process shown in Eq (1).

$$\begin{array}{c} L_{i}^{d}=-\left(y_{i}^{d}\;\text{log}\left(p_{i}\right)+\left(1-y_{i}^{d}\right)\left(1-\text{log}\left(p_{i}\right)\right)\right) \end{array}$$ (1)

where *p*_*i*_ is the probability that the network judges that the video frame is a face. *y*_*i*_ ∈ {0, 1} is the true label of the region.

In addition to determining whether a face is present within a video frame, the face window should be identified as much as possible. In order to achieve this objective, we used the bounding box regression algorithm, with the process using the Euclidean loss function shown in Eq (2).

$$\begin{array}{c} L_{i}^{b}=\|{\hat{y}}_{i}^{b}-y_{i}^{b}{\|}_{2}^{2} \end{array}$$ (2)

where the regression target ${\hat{y}}_{i}^{b}$ is the bounding box coordinates of the network output, and $y_{i}^{b}$ is the true border coordinates, i.e., the quaternion representing the rectangular region.

The predicted feature points are a regression algorithm similar to bounding box regression. This part still used the Euclidean distance as the loss function to calculate the deviation between the predicted feature point coordinates and the actual coordinates, as shown in Eq (3).

$$\begin{array}{c} L_{i}^{m}=\|{\hat{y}}_{i}^{m}-y_{i}^{m}{\|}_{2}^{2} \end{array}$$ (3)

where ${\hat{y}}_{i}^{m}$ is the face feature point coordinates output by the network and $y_{i}^{m}$ is the real feature point coordinates, i.e., the 10-tuple representation of the 5 face feature point coordinates values.

Since there are different training tasks in the three stages, the training images and loss functions are also different in each stage, and the overall learning objective is shown in Eq (4).

$$\begin{array}{c} \text{min}\sum_{i=1}^{N}\sum_{j\in \{d,b,m\}}\alpha_{j}\beta_{i}^{j}L_{i}^{j} \end{array}$$ (4)

where N denotes the number of training samples, i represents the sample label, j shows the task label, and *α*_*j*_ means the importance of the task. $\beta_{i}^{j}\in \left\{0,1\right\}$ is the sample type indicator.

### The proposed CCANet integration framework

**Fig 3:**
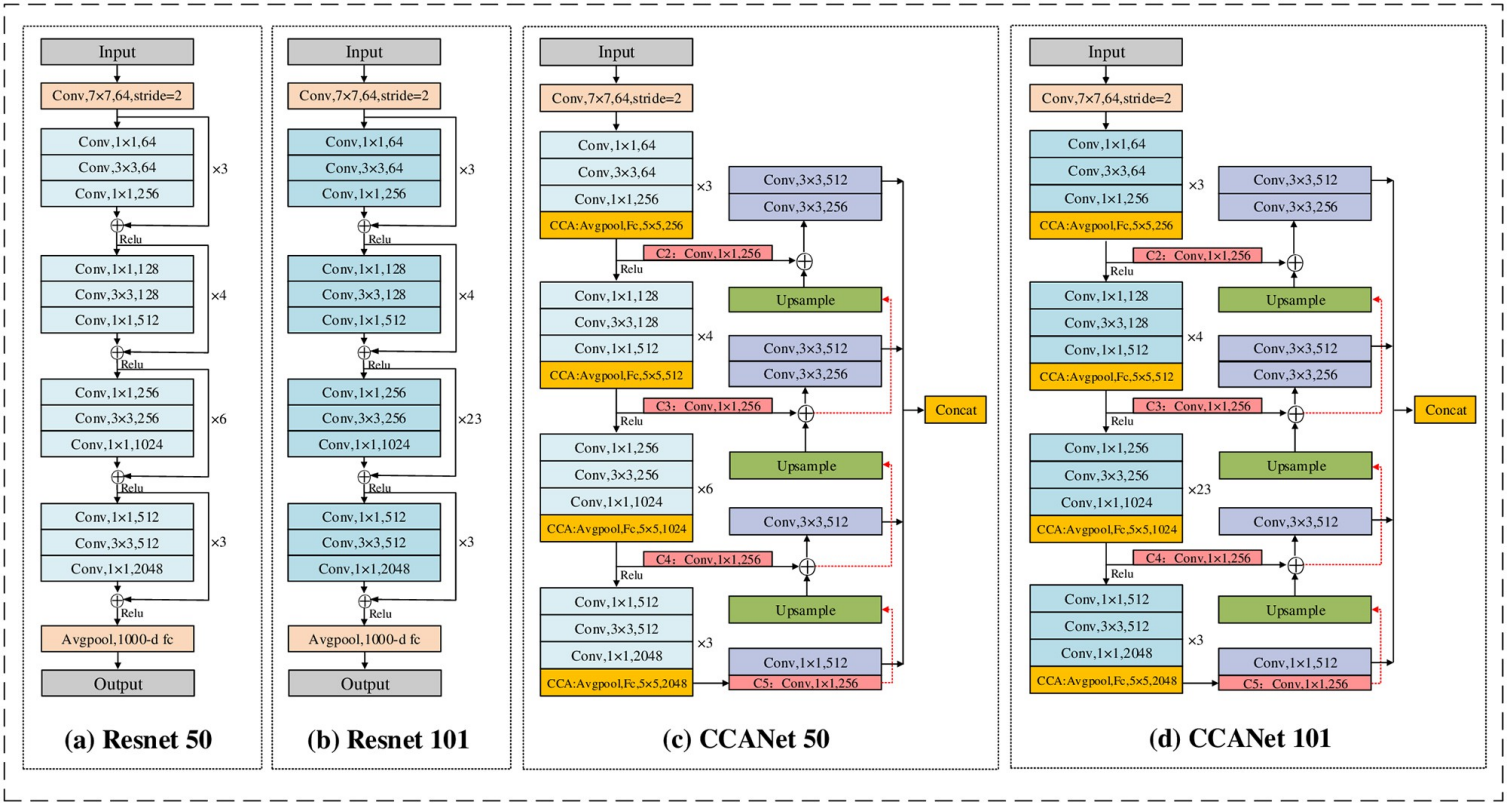
The comparison of Resnet and CCANet structures. (a) shows the structure diagram of Resnet50. (b) is the structure diagram of Resnet101. (c) denotes the structure diagram of CCANet50. (d) represents the structure diagram of CCANet101. The ‘⊕’ indicates that the corresponding elements are summed.

Due to the strong robustness of 2D-CNN in feature extraction, a wide range of applications have been used in computer vision scenarios, such as face recognition and image classification. Therefore, we used the 2D-CNN approach to build a comprehensive framework of CCANet for the automatic diagnosis of depression from videos. The CCANet comprehensive framework consists of two main parts, that is, the FPN module and the CCA-CNN module (called the CCA module in the later sections). The FPN module reduces the loss of minuscule features by enhancing the representation of feature maps of different sizes, and the CCA module is used to enhance the representation between the tensor channels obtained from the convolutional layers to improve recognition accuracy. To further understand the advantages of the CCANet structure, we compared the structures of Resnet50 and Resnet101 with those of our models CCANet50 and CCANet101, as shown in Fig 3. In the following, we described the implementation process of CCANet in detail in terms of two modules, FPN and CCA.

#### Feature Pyramid Networks (FPN) module

**Fig 4:**
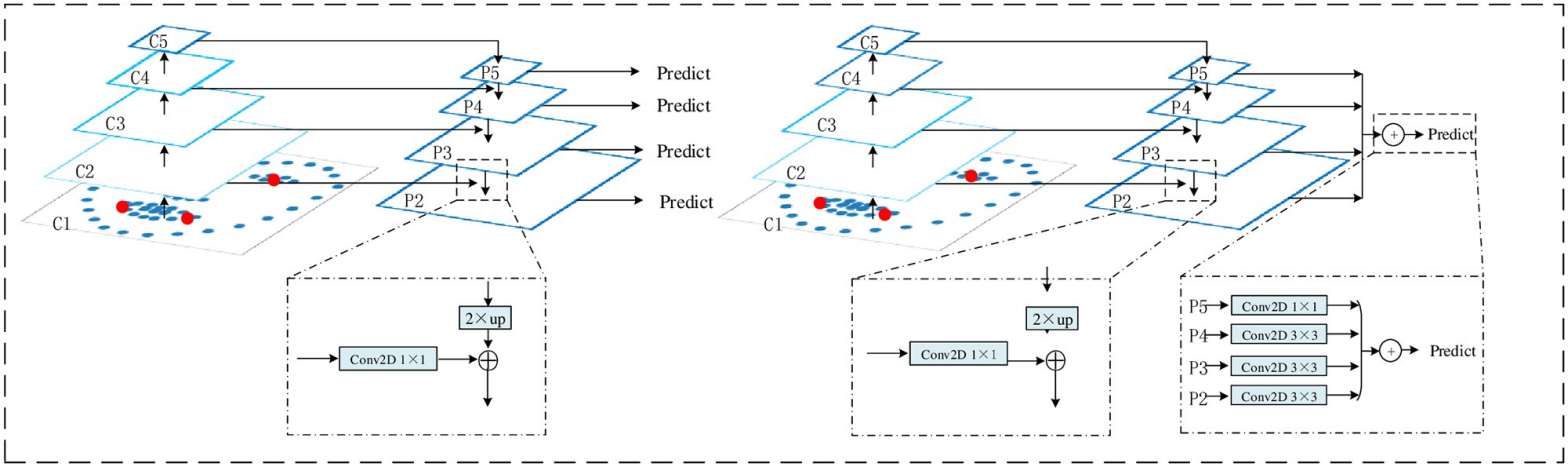
The comparison of the traditional FPN model (left) and the improved FPN model (right) in this paper. The feature map is indicated by the blue outline, and the thicker outline indicates the stronger semantic of the features. The ‘⊕’ indicates that the features are fused by summation.

Currently, existing automatic diagnosis methods for depression ignore the interaction problems between shallow and deep features in face images. In our approach, we introduce the FPN model, which can separate simple target regions using shallow features and complex target regions using deep features. Our method splices the results and finally obtains a face picture with both shallow and deep features, satisfying the needs of face detection and image classification at the same time. The traditional FPN model predicts feature maps of each layer separately, defining the feature maps as {c2, c3, c4, c5}, corresponding to the step size of the original image as {4, 8, 16, 32}. Firstly, each feature map is downsampled in step 2. The size of the input original image is 128 × 128. The original image is downsampled to form an image pyramid {c1, c2, c3, c4, c5} the size of the image {64 × 64, 32 × 32, 16 × 16, 8 × 8, 4 × 4}. The downsampling method used is the nearest neighbor interpolation method, and after the downsampling is finished, the features between images of different sizes are fused by the lateral connection and upsampling process, and the structure is shown in Fig 4. Secondly, the channel number of c5 is changed to 256 by 1 × 1 convolution, and then a 2-fold upsampling is performed using the nearest neighbor interpolation method to obtain the feature map p4, which wide and height is the same as that of c4. Notably, the channel numbers of c4 and p4 are inconsistent, thus c4 is also subjected to 1 × 1 convolution to change its channel number to 256, and then the elements of c4 are summed with p4 individually. The above process is repeated for each of the remaining layers to obtain the respective fused feature vectors. Then the feature vectors obtained from each layer are spliced together for output. The specific splicing process is divided into two main steps. First, the obtained p5 is convolved with convolution kernels of size 1 × 1, and p4, p3 and p2 are convolved with convolution kernels of size 3 × 3, respectively, to form feature maps with the same number of channels. After that, the obtained p5, p4, p3 and p2 are spliced to combine the shallow and deep features of the image to reduce the loss of important features. The spliced feature vectors are fed to the next layer of the network.

The coordinate transformation method of the nearest neighbor interpolation method is shown in Eqs (5) and (6).

$$\begin{array}{c} srcX=dstX\times (srcW/dstW) \end{array}$$ (5)

$$\begin{array}{c} srcY=dstY\times (srcH/dstH) \end{array}$$ (6)

where *dstX* and *dstY* represent the horizontal and vertical coordinates of the target image pixels, *dstW* and *dstH* are the length and width of the target image. *srcW* and *srcH* mean the width and height of the original image, and *srcX* and *srcY* show the coordinates of the original image corresponding to the target image at that point (*dstX*,*dstY*).

#### CCA-CNN Attention Mechanism Module

The neural network can extract the features of the input image to get the corresponding feature maps. The common matrix of the feature map is [C, H, W], while the matrix of the feature map during model training is [B, C, H, W]. Where B denotes the batch size, C represents the channel, H means the height of the feature map, and W is the width of the feature map. In addition, the ability of the network to extract image features can be improved by adding a channel attention mechanism between the convolutional layers when the neural network extracts the image features. Therefore, in our work, we focus on the interactions between tensor channels and propose a new structural unit called the CCA module, which significantly improves the performance of the neural network in extracting image features by increasing the interactions across tensor channels and ensuring the interdependence between tensor channels.

**Fig 5:**
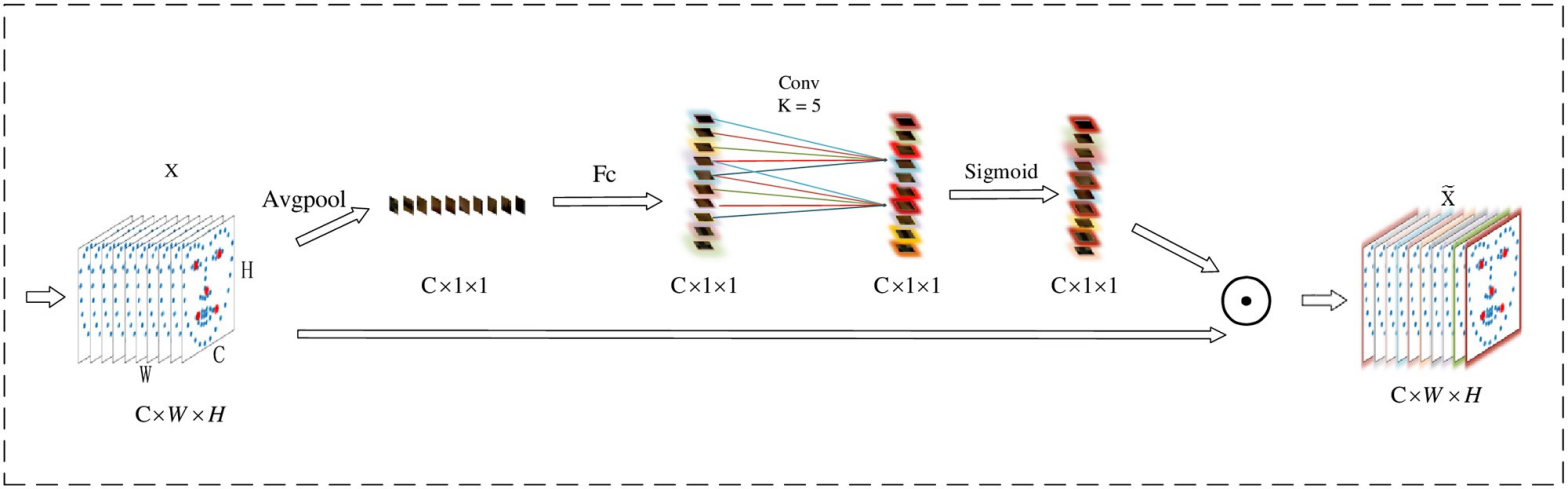
The Attention Mechanism Module of CCA-CNN. C denotes the number of tensor channels of the image. W shows the width of the image. H is the height of the image. Avgpool is the average pooling layer. FC refers to the fully connected layer. k = 5 denotes a convolution with a convolution kernel size of 5. Sigmoid is the activation function, and ‘⊙’ denotes the phase multiplication.

As shown in Fig 5, the general idea of the CCA module is that each tensor channel of the feature map has its weight, so different tensor channels have different effects on the extracted features. Starting from an image with input feature dimension [C, H, W], the [H, W] dimension of the feature map goes through the averaging pooling layer to obtain a feature map of size [C, 1, 1]. We regard the global averaging pooling, the input to the averaging pooling layer in the structure is X and is the result of performing global averaging pooling on the feature map X in the spatial dimension. Since facial features are relatively subtle and easily affected by changes in the regions around the point coordinates, if maximum pooling is used, some changes in the nearby regions will be ignored, resulting in feature loss, while average pooling will fully consider the combined changes in the nearby regions and the target region, making it easier to reflect the differences in the features. Therefore, each element of z is shown in Eq (7).

$$\begin{array}{c} Z_{c}=F_{A}\left(X\right)=\frac{1}{H\times W}\sum_{i=1}^{H}\sum_{j=1}^{W}X\left(i,j\right) \end{array}$$ (7)

where *X* = [*x*^1^, *x*^2^, *x*^3^, ⋯, *x*^*c*^], *z*_*c*_ ∈ *R*^*C*^.

The average pooled vector is passed through a layer of fully connected neural network with the aim of capturing the dependencies of the channels on the image features. To achieve this goal, we chose a simple gating mechanism with a sigmoid activation function. For the input of the fully connected layer, the output is shown in Eq (8).

$$\begin{array}{c} S=F_{c}\left(z,W\right)=\sigma \left(g\left(z,w\right)\right)=\sigma \left(W_{2}\delta \left(W_{1}z\right)\right) \end{array}$$ (8)

where *S* denotes the output of the fully connected layer, *σ* represents the sigmoid activation function, *δ* shows the ReLu activation function, $W_{1}\in R^{\frac{C}{r}\times C}$, $W_{2}\in R^{\frac{C}{r}\times C}$, the value of *r* in this paper is 16, which represents the decay rate.

The output S of the last fully-connected layer is input to a one-dimensional convolutional neural network with a convolutional kernel size of 5 to achieve cross-channel interaction, which ensures the efficiency of feature extraction, and the output of S after input to the convolutional layer is shown in Eq (9).

$$\begin{array}{c} \omega =\sigma (C1D_{k}\left(S\right)) \end{array}$$ (9)

where *ω* denotes the output of the convolution layer, *σ* denotes the sigmoid activation function, and *C*1*D*_*k*_ denotes a one-dimensional convolution with *k* = 5 convolution kernel size.

Finally, the final output of the module is obtained by rescaling the feature map using the activation method to transform the output X, and the activation method is shown in Eq (10).

$$\begin{array}{c} \tilde{X}=F_{s}\left(x_{c},\omega_{c}\right)=x_{c}\omega_{c} \end{array}$$ (10)

where $\tilde{X}=\left[\tilde{x_{1}},\tilde{x_{2}},\cdots ,\tilde{x_{c}}\right]$, *x*_*c*_ ∈ *R*^*H*×*W*^, *F*_*s*_ is the channel-wise multiplication between the feature map *x*_*c*_ and the channel weights *ω*_*c*_.

## Experiments and results

### AVEC 2014 depression dataset

In this section, we demonstrate the feasibility of CCANet by conducting experiments of different sizes on the publicly available depression detection dataset AVEC 2014.

In our study, all experiments have been verified on the publicly available AVEC 2014 dataset for depression, which was provided for the 2014 audio/visual emotion challenge [18]. The age of the subjects ranged from 18 to 63 years with an average age of 31.5 years. The dataset contains two tasks, “Freeform” and “Northwind”, in which subjects were asked to answer a series of questions or describe a sad childhood memory using the German language in the “Freeform” task, while they were asked to read an excerpt from the German fable “The North Wind and the Sun” in the “Northwind” task. In these two tasks, 150 video clips of 82 subjects were recorded by using a webcam and microphone to record the appearance signal and audio of subjects performing a human-computer interaction task in a quiet environment. In these video clips, some subjects appeared in multiple clips, but only one person appeared in each clip, and the length of each video clip varied from 6 seconds to 4 minutes. AVEC 2014 dataset is divided into the training set, the development set, and the test set, among which each set contains 100 samples. The Becker Depression Inventory-II (BDI-II) [7, 17] was used to mark the level of depression in the AVEC 2014 dataset.

We trained our CCANet model on the AVEC 2014 dataset with the training set and then used the validation set to adjust the various parameters in the experiment and verify the effectiveness of each module, after which we used the test set to determine the applicability of the CCANet model and to compare the test results with the existing results for analysis.

### Experimental setup and evaluation measures

#### Experimental setup

All of our experiments were conducted on the deep learning framework PyTorch. Due to the high temporal redundancy of the AVEC 2014 dataset, the videos need to be frame-separated to reduce the total number of images for each video input and make the images more representative. 100-105 frames are extracted from each video separately, depending on a fixed time interval. The face key points are detected and cropped for each video frame by the MTCNN, and then the video frame is resized to 128 × 128 according to the face key point coordinates for the input of the neural network.

After the above processing, we perform the representation of face image sequence features for each video frame in the dataset, and then randomly sample from each video frame and input to the CCANet network with one batch of every 128 frames to complete the training. The Mean Square Loss function (MSELoss) was used in the training process, and the ReLU was used as the activation function for all layers in the depression detection model. In addition, the Adam optimizer is used in the training and has an initial learning rate of 0.001, a decay rate of 0.0001, and the ratio of the training set to the validation set of 3:1. Finally, the model saves the result with the least loss in the validation set among 100 training times.

The loss function MSELoss of the training process is shown in Eq (11).

$$\begin{array}{c} loss\left(x_{i},y_{i}\right)={\left(x_{i}-y_{i}\right)}^{2} \end{array}$$ (11)

where *loss*, *x*, and *y* have the same dimensions and can be vectors or matrices, and *i* is the subscript.

#### Evaluation metrics (RMSE, MAE)

Currently, Root Mean Square Error (RMSE) and Mean Absolute Error (MAE) have been widely used as measures of diagnostic accuracy for depression severity. At the time of the AVEC 2014 dataset release, RMSE and MAE were used to measure the experimental performance of the given baseline method. To make a fair comparison, RMSE and MAE are still used as measures in this paper, with their calculations shown in Eqs (12) and (13), respectively.

$$\begin{array}{c} RMSE=\sqrt{\frac{1}{N}\sum_{i=1}^{N}{\left(y_{i}-\hat{y_{i}}\right)}^{2}} \end{array}$$ (12)

$$\begin{array}{c} MAE=\frac{1}{N}\sum_{i=1}^{N}|y_{i}-\hat{y_{i}}| \end{array}$$ (13)

where *N* is the number of subjects, *y*_*i*_ is the BDI-II given by the data set, and $\hat{y_{i}}$ denotes the BDI-II predicted scores obtained by the experimental procedure.

From the formula, the closer the predicted BDI-II scores are to the true values, the smaller the values of these two metrics are, and the better the experimental performance obtained. Therefore, it is reasonable by using RMSE and MAE to measure the experimental performance of prediction algorithms with different degrees of depression.

#### Ablation experiment

**Fig 6:**
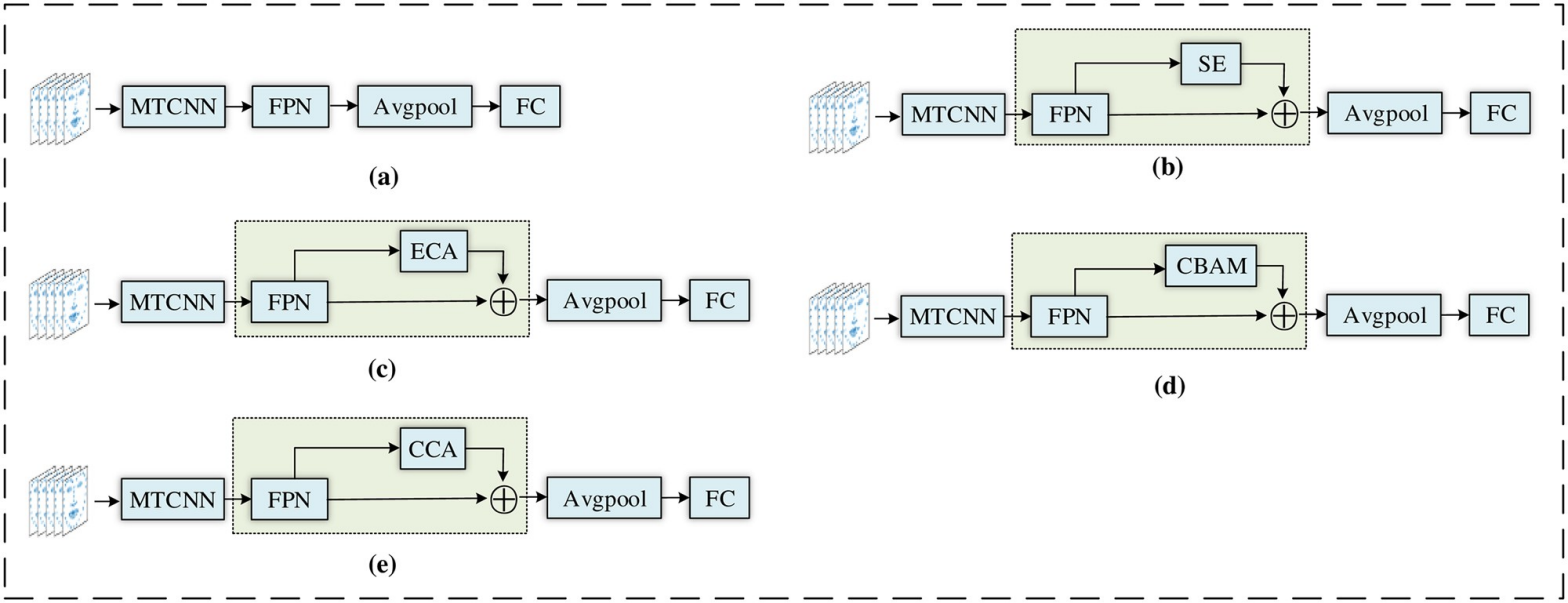
The network model for conducting ablation experiments. (a) refers to the network structure of the FPN module. (b) represents the network structure with the SE attention mechanism included in the FPN module. (c) shows the network structure with the ECA attention mechanism included in the FPN module. (d) is the network structure with the CBAM attention mechanism included in the FPN module. (e) is the network model of CCANet.

In this paper, we tested the effectiveness of the CCA module by designing five combinations of FPN, FPN + SE, FPN + ECA, FPN + CBAM and FPN + CCA on the AVEC 2014 depression dataset for ablation experiments, as shown in Fig 6. Among them, the SE attention mechanism [11, 47, 48], the ECA attention mechanism [49], and the CBAM attention mechanism [48, 50] have been shown to be effective in depression detection studies. Training and testing were performed under the same settings and the corresponding experimental results are shown in Table 2. The RMSE and MAE obtained by using the FPN module alone are 10.98 and 8.86, respectively, which indicates that using the FPN module alone does not achieve depression recognition. Compared with adding only the FPN module, the RMSEs obtained by adding only the SE module the ECA module, or the CBAM module were 9.73, 9.38 and 9.24 respectively, and the MAEs obtained were 7.52, 7.45 and 7.38 respectively, which suggests that using the Attention Mechanism Module is effective in improving the accuracy of detection. In the experiments using our proposed CCA module, the best results for RMSE and MAE are 8.65 and 6.66 respectively, which suggests that the CCA module has improved fitting ability in the field of depression recognition.

**Table 2:** Comparison of the prediction results using different networks on the AVEC2014 dataset.

Models	RMSE	MAE
FPN	10.98	8.86
FPN+SE	9.73	7.52
FPN+ECA	9.38	7.45
FPN+CBAM	9.24	7.38
Ours/CCANet50	9.19	7.03
Ours/CCANet101	8.65	6.66

#### Experimental results

**Fig 7:**
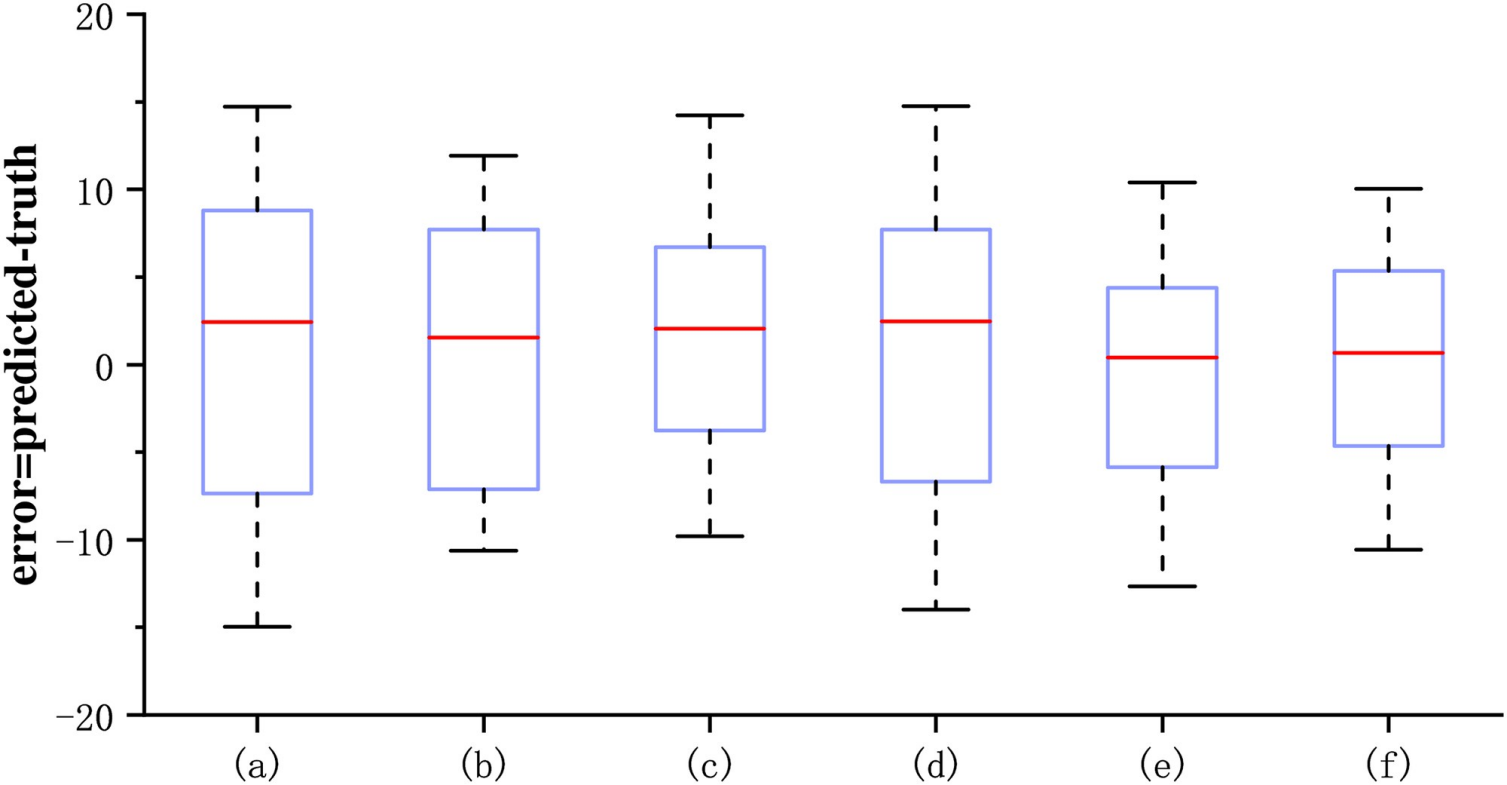
The box plots of the prediction errors for different models on the AVEC 2014 depression dataset. (a) refers to the network structure of the FPN module. (b) represents the network structure with the SE attention mechanism included in the FPN module. (c) shows the network structure with the ECA attention mechanism included in the FPN module. (d) is the network structure with the CBAM attention mechanism included in the FPN module. (e) is the network model of CCANet50. (f) is the network model of CCANet101.

**Fig 8:**
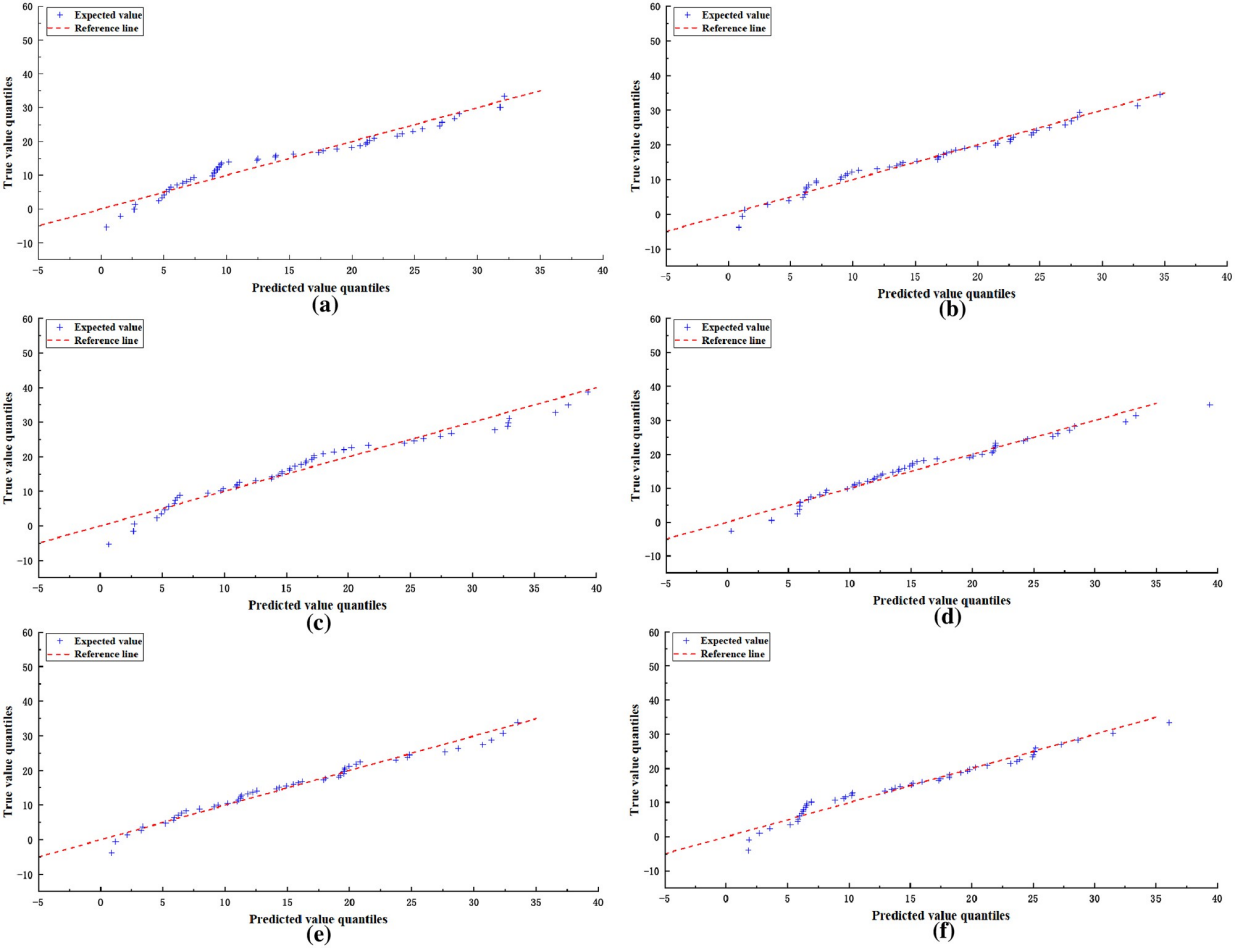
The Q-Q plots of different models on the AVEC 2014 depression dataset. (a) refers to the network structure of the FPN module. (b) represents the network structure with the SE attention mechanism included in the FPN module. (c) shows the network structure with the ECA attention mechanism included in the FPN module. (d) is the network structure with the CBAM attention mechanism included in the FPN module. (e) is the network model of CCANet50. (f) is the network model of CCANet101.

To illustrate the superiority of the CCA module, we produced box plots of prediction errors for different models and Q-Q plots of predicted versus true values, respectively, which can visualize the degree of data dispersion and the homogeneity of the data to judge the validity of the results. As shown in Fig 7, the heights of e and f are the smallest in the boxplot, which represents that the predicted data of e and f are less volatile, c,e, and f have a smaller quartile range, which indicates that the predicted data are relatively concentrated, from the median (the red line in the figure), the data of e and f are closer to 0, which represents that the error between predicted value and true value of e and f is less, thus judging that CCANet50 and CCANet101 have low discretization and high data homogeneity. Fig 8 mainly judges whether the deviation of the predicted data conforms to the normal distribution, as can be seen from the figure, the horizontal and vertical coordinates represent the predicted value and the true value respectively, and the red dotted line in the figure is y = x, which indicates that when the predicted data is closer to the true value the distribution of the data is closer to y = x, and it can be seen that the data of e can be uniformly distributed on y = x, following by f, which indicates that the distribution of the prediction result of our model is more uniform and can obey the normal distribution. We can conclude that CCANet has achieved comparability in improving the prediction accuracy of BDI-II, which validates the effectiveness of the CCA module in depression detection.

The proposed framework achieves comparable performance to most state-of-the-art methods, and as a comprehensive framework, CCANet can learn facial behaviors better than traditional methods and capture visual features with variations efficiently, which is important for the automatic learning of facial expression coding features with inference capability. As shown in Table 3, the literature [18, 24, 35, 36, 51] uses methods based on manual creation of descriptors such as support vector regression, partial least squares, LPQ, LBP, etc. The SVM algorithm solves support vectors with the help of quadratic programming, similar to the SVM algorithm, and partial least squares are only suitable for regression modeling where the number of samples is less than the number of independent variables, which makes it difficult to be implemented on a large scale. Algorithms such as local binary patterns and local phase quantization are mainly based on the multi-fusion of underlying features such as color, texture, shape, etc. The disadvantages of these algorithms are that the models are more complex and are easily affected by factors such as illumination, image rotation, low resolution, etc., thus reducing the recognition accuracy. Unlike them, our trained deep neural network outperforms their methods in terms of efficiency and accuracy, this is mainly because hand-crafted features can only depict depression cues from a single aspect and are profoundly dependent on the experience of the designer.

**Table 3:** The comparison of depression severity diagnosis with different methods on the AVEC 2014 depression dataset.

Methods	RMSE	MAE
Valstar et al.(2014)/LGBP-TOP,SVR [18]	10.86	8.86
Kaya et al.(2014)/LGBP-TOP,LPQ [24]	10.26	8.20
Dhall et al.(2015)/LBP-TOP,SVR [51]	8.91	7.08
He et al.(2018)/MRLBP-TOP,SVR [35]	9.01	7.21
Niu et al.(2019)/LSOGCP-TOP [36]	9.10	7.19
He, Guo, Tiwari.(2022)/2D-CNN [11]	9.03	7.26
Zhu et al.(2017)/2D-CNN [37]	9.55	7.47
Uddin et al.(2020)/LSTM [42]	8.78	6.86
Zhou et al.(2020)/2D-CNN [52]	**8.30**	**6.59**
Niu et al.(2020)/2D-CNN [53]	8.81	6.72
Al Jazaery et al.(2018)/C3D,RNN [39]	9.20	7.22
de Melo et al.(2019)/C3D [41]	**8.31**	**6.59**
He, Guo et al.(2022)/3D-CNN [54]	8.42	6.78
**Ours/CCANet**	**8.65**	**6.66**

Literature [11, 37, 42, 52, 53] et al adopted the 2D-CNN approach to reduce the incompleteness caused by human-designed features and achieved relatively high accuracy in depression recognition. Uddin et al. [42] introduced LSTM to obtain temporal information by integrating temporal median pools, however, LSTM has more parameters, which increases the training time of the model and is not conducive to the training of large-scale data. He et al. [11] proposed a comprehensive framework for the automatic diagnosis of depression based on video image sequences, adopted multiple pre-trained models to represent the low-level features, and proposed a feature aggregation module to capture the high-level features, our comprehensive framework introduced the FPN pyramid model, which can efficiently extract the different levels of semantic features, and proposed a CCA module to enhance the characterization of the facial feature points, and the results obtained the RMSE was improved by 0.38, and the MAE was improved by 0.6.

Literature [39, 41, 54] has used a 3D convolutional neural network approach, and although the 3D convolutional neural network approach has also obtained relatively good accuracy in depression recognition, the model complexity of 3D convolutional neural networks is higher than that of 2D convolutional neural networks, which increases the computational volume and training time of model training, and imposes stricter requirements on the performance of computers. Zhou et al. [52] and de Melo et al. [41] required fine-tuning of the model from the facial images to the AVEC 2014 large pre-trained deep models on the database with slightly higher accuracy than our test results, the reason may be that the pre-trained deep models contain typical features that simulate the severity of depression, which reduces the cost of training the model, whereas our approach is a complete end-to-end depression recognition scheme from data analysis and processing, model training to accuracy calculation, which does not require the use of a pre-trained model to recognize depression. In contrast, our model reduces model complexity, saves training time, and obtains competitive results.

Overall, our model selected five facial key points of the participant’s eyes, corners of the mouth and tip of the nose, effectively extracted to different layers of semantic features by employing the FPN pyramid, and combined with the CCA module to enhance the characterization ability of the facial feature points and interactions between the tensor channels, which achieved higher accuracy in the field of depression recognition, further proving the superiority of the model we designed in the work of depression level detection.

## Conclusion

In this paper, we develop an automated depression detection system called CCANet, which can recognize facial expressions from recorded video sessions of depressed patients and detect the changing characteristics of key points on the face to analyze the depression level of the subjects. In this system, we used a deep learning approach to extract facial features of images to generate feature vectors that were applied to video samples. Subsequently, we used regression to correlate facial features and depression levels and performed extensive experiments on the AVEC 2014 depression dataset yielding RMSE = 8.65 and MAE = 6.66, demonstrating the validity of the model. During the development of the model, we highlight three main contributions. The first contribution is that we improved the FPN model by fusing shallow and deep features, reducing the loss of miniscule features and improving the feature extraction capability. The second contribution is our proposed deep feature extraction method, which eliminated the drawbacks of traditional manual feature extraction methods and improved the accuracy of depression recognition by exploiting key point features of faces in images. The third contribution is the proposed CCA module, which enhances the characterization ability of the facial feature points and interactions between the tensor channels. The method is proposed and applied to help improve the diagnostic accuracy of physicians in depression clinics, and also help patients to detect the condition and take interventions in time by establishing a depression testing and online treatment system to further reduce the risk of the disease.
